# A rare presentation of pulmonary sarcoidosis as a solitary lung mass: a case report

**DOI:** 10.1186/s13256-018-1632-0

**Published:** 2018-04-13

**Authors:** Dylan W. Kelleher, Madeleine Yaggi, Robert Homer, Erica L. Herzog, Changwan Ryu

**Affiliations:** 1 Department of Internal Medicine, Section of Pulmonary, Critical Care and Sleep Medicine, Yale University School of Medicine, 300 Cedar Street, TAC 441 South, P.O. Box 208057, New Haven, CT 06520 USA; 2 Department of Pathology, Yale University School of Medicine, New Haven, CT USA

**Keywords:** Pulmonary sarcoidosis, Lung mass, Sarcoid-like reaction

## Abstract

**Background:**

Sarcoidosis is a multisystem, chronic granulomatous disease of unknown etiology that predominantly affects the lungs. Pulmonary sarcoidosis classically presents with constitutional symptoms and computed tomographic scan findings of bilateral, symmetric micronodules in a peribronchovascular distribution with upper and middle lung zone predominance accompanied by bilateral, symmetric hilar lymphadenopathy. A solitary lung mass is a rare finding for pulmonary sarcoidosis, and with its associated constitutional symptoms, it strongly mimics a malignancy. We aimed to provide further insight into the broad differential diagnosis of a lung mass by describing our experiences in the care of a patient who presented with clinical and radiographic features of lung cancer who was ultimately found to have an atypical manifestation of stage II pulmonary sarcoidosis.

**Case presentation:**

A 44-year-old African American woman with a history of childhood asthma and type 2 diabetes mellitus presented with shortness of breath. After being treated for a presumed asthma exacerbation with prednisone, she experienced worsening dyspnea, night sweats, and unintentional weight loss. Further evaluation revealed a large left lower lobe mass and hilar lymphadenopathy. A computed tomography-guided biopsy of the lung mass revealed a multifocal non-necrotizing granuloma with multinucleated giant cells. Although consistent with sarcoidosis, this finding could represent a sarcoid-like reaction secondary to an occult malignancy. A more extensive repeat biopsy via bronchoscopy and mediastinoscopy revealed granulomatous inflammation without evidence of malignancy or infection. These procedures confirmed the diagnosis of pulmonary sarcoidosis, and she was started on treatment with high-dose prednisone. Her treatment course was complicated by hyperglycemia necessitating insulin therapy, but after 3 months of therapy, she reported improvement in her dyspnea, and repeat imaging revealed a significant decrease in the size of the lung mass and lymphadenopathy. Given her clinical and radiographic response, she was continued on a prednisone taper.

**Conclusions:**

Atypical manifestations of pulmonary sarcoidosis are diagnostically challenging because the clinical and radiographic features of the disease mimic those of a malignancy. We aimed to illustrate a unique etiology of a lung mass and the importance of maintaining a broad differential diagnosis. Nonetheless, with the possibility of a malignancy, a high index of suspicion is necessary for timely diagnosis and optimal management.

## Background

Sarcoidosis is a systemic, chronic granulomatous disease of unknown etiology that commonly affects the lungs [1]. Whereas some patients with pulmonary sarcoidosis are asymptomatic, many report cough, dyspnea, fatigue, unintentional weight loss, and night sweats [2]. Because of its nonspecific symptomology, computed tomographic (CT) scans are instrumental for diagnosis [1, 2]. Common findings include bilateral, symmetric micronodules in a peribronchovascular distribution with upper and middle lung zone predominance accompanied by bilateral, symmetric hilar lymphadenopathy [3]. However, atypical radiographic patterns of pulmonary sarcoidosis [3] prove to be diagnostically challenging, particularly in the rare cases of mass lesions, because the associated constitutional symptoms strongly mimic a malignancy [4]. We aimed to provide further insight into the broad differential diagnosis of a lung mass by describing our experiences in the care of a patient who presented with clinical and radiographic features of lung cancer who was ultimately found to have a rare manifestation of stage II pulmonary sarcoidosis.

## Case presentation

A 44-year-old African American woman with a past medical history significant for childhood asthma and type 2 diabetes mellitus presented to our institution with dyspnea. One month prior to presentation, she complained of shortness of breath that was presumed to be due to an asthma exacerbation and was treated with an empiric, 2-week course of prednisone. She noted that her breathing improved while on prednisone, but she returned to our emergency department with worsening dyspnea once prednisone was tapered off. Her review of systems revealed night sweats for 2 months and an unintentional weight loss of 15 pounds.

**Fig. 1:**
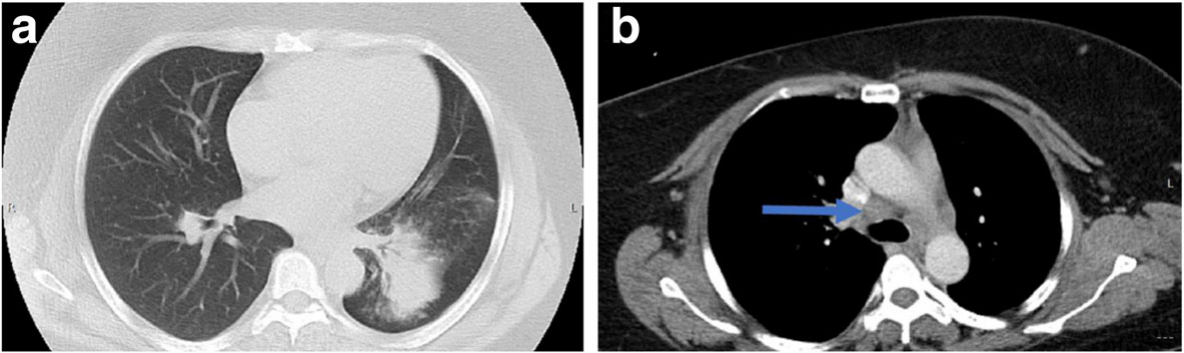
Chest computed tomography reveals a large left lower lobe lung mass. On admission, the patient was found to have (**a**) a 6.7 × 5.4 × 9.9-cm left lower lobe mass and (**b**) hilar lymphadenopathy (*arrow*)

**Fig. 2:**
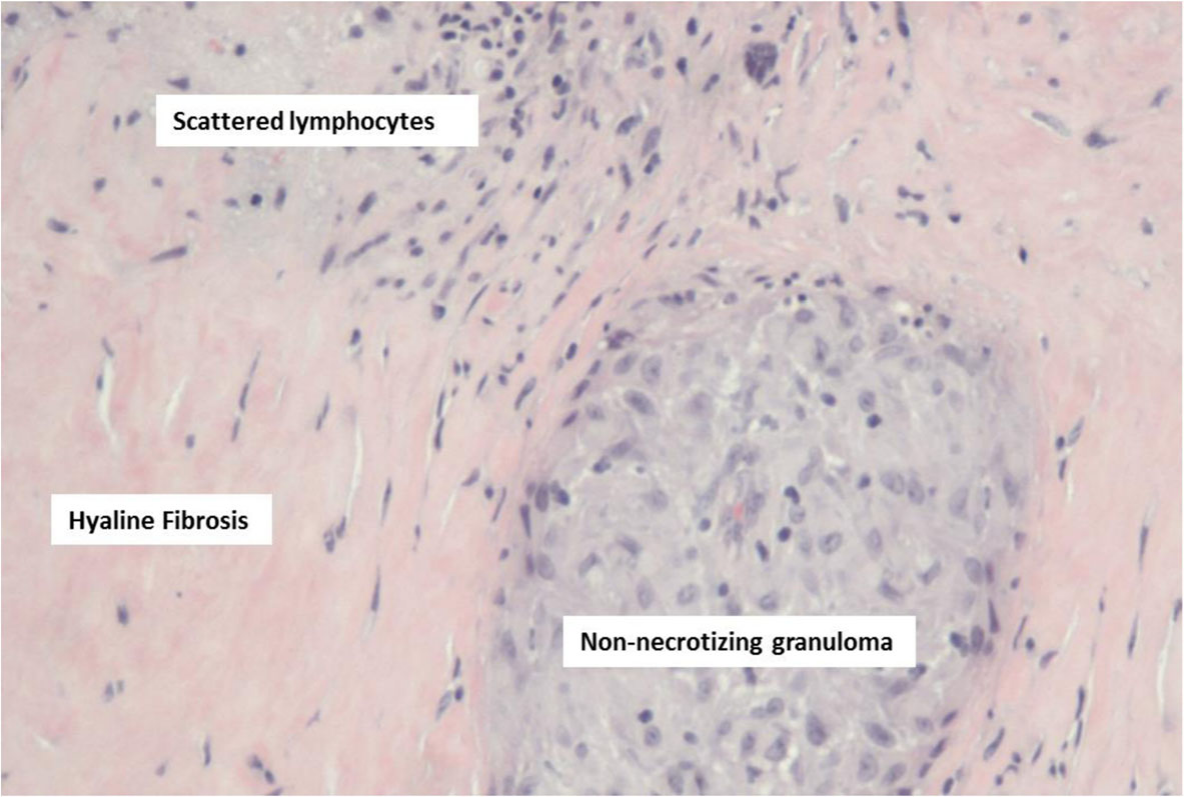
Biopsy of hilar lymph node revealed granulomatous inflammation. High-power view of the right hilar lymph node (station 4R) biopsy shows non-necrotizing granuloma with scattered lymphocytes and surrounding fibrosis

Her vital signs and results of her physical examination were normal. Laboratory testing revealed normal complete blood count, chemistry panel, calcium, and vitamin D values. A chest radiograph revealed a masslike opacity in the left lower lobe, which was followed with a chest CT scan that revealed a large, 6.7 × 5.4 × 9.9-cm left lower lobe mass (Fig. 1a) and hilar lymphadenopathy (Fig. 1b). Given the patient’s ongoing constitutional symptoms, there was significant concern for a pulmonary malignancy; the mass was felt to be too distal for transbronchial biopsies via bronchoscopy, and a computed tomography-guided biopsy of the lung mass was performed, which revealed multifocal non-necrotizing granulomas with multinucleated giant cells without evidence of malignancy or active infection. Although the biopsy was consistent with sarcoidosis, this finding could represent a sarcoid-like reaction secondary to an occult malignancy. Subsequently, a repeat biopsy of the mass was successfully attempted via transbronchial biopsy via bronchoscopy, and a mediastinoscopy was performed to obtain sufficient tissue for a definitive diagnosis. The lung mass biopsy was again consistent with granulomatous inflammation without evidence of malignancy or active infection, and the lymph node biopsy (station 4R) revealed active granulomatous inflammation (Fig. 2).

**Fig. 3:**
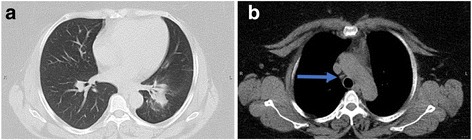
Repeat chest computed tomography revealed a decrease in the size of the lung mass and lymphadenopathy. Following 3 months of high-dose prednisone therapy, repeat computed tomographic scan revealed a significant decrease in (**a**) the size of the lung mass, which measured 4.7 × 3.1 × 5.8 cm, and (**b**) lymphadenopathy (*arrow*)

Following these two procedures, we were confident in our diagnosis of pulmonary sarcoidosis. Given her debilitating symptoms, she was started on treatment with prednisone 60 mg daily with appropriate prophylaxis. Within 1 month of treatment, she began to experience improvement of her dyspnea; however, her glucose control deteriorated to a point where she required insulin to manage her diabetes. A repeat CT scan after 3 months of high-dose prednisone revealed a significant decrease in the size of the lung mass, which measured 4.7 × 3.1 × 5.8 cm (Fig. 3a), and lymphadenopathy (Fig. 3b). Although we considered starting a steroid-sparing agent, we continued her on a prednisone taper, given her robust clinical and radiographic response.

## Discussion

Atypical radiographic presentations of pulmonary sarcoidosis occur in 15–25% of patients [3]. These findings can include diffuse ground-glass opacities, honeycombing, multiple nodules, or necrotizing consolidations [3]. A large, solitary lung mass, as described in our patient’s case, has rarely been reported, and its incidence is unknown; it is likely to be the result of individual granulomas that have coalesced to produce the appearance of a mass [5].

Because solitary lung masses with mediastinal lymphadenopathy are the hallmarks of lung cancer, obtaining a definitive diagnosis in cases of atypical pulmonary sarcoidosis is diagnostically challenging. Prior case reports described the need for a surgical lung biopsy to obtain sufficient tissue for a definitive diagnosis [4, 6], which was the case in our patient. However, the presence of granulomas does not necessarily rule out a malignancy, because non-caseating granulomas have been reported in some instances of small cell lung cancer [7]. Moreover, the presence of non-caseating granulomas in association with a malignancy, described as a sarcoid-like reaction, has been shown to occur adjacent to the site of the malignancy or around local lymph nodes [7, 8]. Sarcoid-like reactions, which are histologically indistinguishable from granulomas found in usual cases of sarcoidosis, are believed to be the result of an immunological response to local tumor products [8]. They occur in the context of both hematologic malignancies and solid tumors; one study demonstrated an association between sarcoid-like reactions and lung cancer [8]. In addition, sarcoid-like reactions occur in response to microbial infection, such as leishmaniasis, tuberculosis, and coccidioidomycosis [9]. Thus, in cases of atypical sarcoidosis, biopsies from two noncontiguous sites are recommended to rule out an active malignancy or infection [10].

Although prednisone is the first-line treatment for sarcoidosis [1, 2], in cases with significant adverse reactions, such as the one presented here, steroid-sparing agents can be considered. Methotrexate is a commonly chosen second-line agent because it has been shown to be effective in decreasing the necessary dose of prednisone when used in combination [11]. For refractory cases, leflunomide and tumor necrosis factor-α antagonists, such as infliximab, adalimumab, or etanercept, have demonstrated efficacy in small observational studies [11, 12].

## Conclusions

Atypical manifestations of pulmonary sarcoidosis are diagnostically challenging because the clinical and radiographic features of the disease mimic those of a malignancy. We aimed to illustrate a unique etiology of a lung mass, the importance of maintaining a broad differential diagnosis, and the need for a thorough evaluation. Nonetheless, with the possibility of an active malignancy, a high index of suspicion is necessary for timely diagnosis and optimal management.
